# Is Speciation Accompanied by Rapid Evolution? Insights from Comparing Reproductive and Nonreproductive Transcriptomes in *Drosophila*

**DOI:** 10.4061/2011/595121

**Published:** 2011-08-22

**Authors:** Santosh Jagadeeshan, Wilfried Haerty, Rama S. Singh

**Affiliations:** ^1^Department of Biology, McMaster University, Hamilton, ON, Canada L8S 4KI; ^2^Smithsonian Tropical Research Institute, P. O. Box 0834-03092, Balboa, Ancón, Panama

## Abstract

The tempo and mode of evolutionary change during speciation have remained contentious until recently. While much of the evidence claiming speciation is an abrupt and rapid process comes from fossil data, recent molecular phylogenetics show that the background of gradual evolution is often broken by accelerated rates of molecular evolution during speciation. However, what kinds of genes affect or are affected by speciation remains unexplored. Our analysis of 4843 protein-coding genes in five species of the *Drosophila melanogaster* subgroup shows that while ~70% of genes follow clock-like evolution, between 17–19.67% of loci show signatures of accelerated rates of evolution in recently formed species. These genes show 2-3-fold higher rates of substitution in recently diverged species compared to older species. This fraction of loci affects a diverse range of functions. Only a small proportion of reproductive genes experience speciation-related accelerated changes but many sex-and -reproduction related genes show an interesting pattern of persistent rapid evolution suggesting that sex-and-reproduction related genes are under constant selective pressures. The identification of loci associated with accelerated evolution allows us to address the mechanisms of rapid evolution and speciation, which in our study appears to be a combination of both selection and rapid demographical changes.

## 1. Introduction

The tempo and mode of evolutionary change during speciation have remained a contentious issue for more than five decades. Evidence for abrupt and rapid changes during speciation came from fossil evidence but lacked mechanistic explanations for such a process [1–7]. Since the mid-1970s, molecular phylogenetic studies began to associate increased genetic changes with speciation events suggesting that rates of molecular evolution might be altered during speciation [8–11]. This trend has been recently confirmed by molecular phylogenies using large numbers of genes and a wide variety of taxa, explicitly showing that speciation is accompanied by accelerated rates of molecular evolution [12–15]. 

What remains unknown is the mode of change during speciation, that is, the kind of genetic changes associated with speciation. More specifically, the numbers and kinds of protein coding genes that change during speciation remain unexplored. Accelerated evolution is only observed in a fraction of genes analyzed by Pagel et al. [13] as well as in other genome-wide estimates of molecular divergences [16–18]. A more systematic search to identify protein-coding genes that experience accelerated evolution during speciation will allow us to directly address the mode of evolutionary change during speciation, that is, divergences, in what kinds of genes, affect or are affected by speciation. 

Taxa-wide evidence of sex- and reproduction-related genes evolving rapidly in sibling species [19, 20] has raised the possibility that sex-related genes might preferentially experience elevated rates of evolution during the speciation process, or may even drive speciation by causing reproductive isolation among diverging populations. This framework is supported by the fact that almost all candidate “speciation” genes identified, so far, are mainly sex related (with the exception of a few genes with other functions), evolve rapidly between closely related species, often show signatures of adaptive evolution and have been invoked in the rapid evolution of hybrid sterility in different organisms [21–32]. After controlling for incomplete lineage sorting in the melanogaster subgroup, we put this framework to test by analyzing 4843 protein coding genes in the Drosophila transcriptome for signatures of speciation-related accelerated changes.

Divergence trends between recently formed species and old species provide a proxy to detecting signatures of speciation-related accelerated changes. Molecular divergences are expected to be proportional to the duration of a species' existence; newly formed species will have accumulated less molecular divergence compared to older species. Signatures of speciation-related accelerated evolution will be manifested as relatively higher rates of molecular evolution in newly formed species compared to species with longer postspeciation divergence times. Accordingly we asked the following questions: (1) are rates of molecular evolution in protein-coding genes affected by speciation events? That is, do genes show unexpectedly elevated rates of evolution in newly formed species relative to older species? (2) do sex-related genes preferentially exhibit accelerated speciation-related changes relative to non-sex related genes? And (3) do genes with accelerated rates of evolution show evidence of positive selection?

## 2. Methods

### 2.1. Rationale

If evolutionary rates of protein coding genes are not affected by speciation and evolve at a constant rate, we should be able to find a correlation between the length of time that species have diverged and the proportion of molecular divergence between these species. Species diverged for longer lengths of time would have accumulated proportionally higher amounts of genetic changes relative to species diverged for shorter periods of time. Conversely, if rates of molecular evolution were indeed affected by speciation, then this correlation will be broken and newer species might show relatively higher evolutionary rates compared to older species. Our analyses therefore primarily exploit the nature of molecular changes *since* divergence of species pairs (recent versus older) to infer speciation-related accelerated changes.

### 2.2. The Phyletic System

We used a phyletic system comprised of three pairs of closely related species from the melanogaster subgroup* D*. *simulans *and *D. sechellia* diverged about 0.3–0.6 Mya [33], *D. melanogaster* and *D. simulans* diverged about 4.3–6.5 Mya, and *D. yakuba* and *D. erecta* diverged about 8.1–12.7 Mya [34]. Divergence times for *D. simulans-D. melanogaster* and *D. yakuba-D. erecta* were recently reestimated by using over 2977 nuclear genes and by implementing a novel genomic-mutation distance approach correcting for codon bias [34] whereas the divergence times for the *D. simulans-D. sechellia* split were estimated using a small number of genes [33]. All of these divergence dates broadly concur with other independent estimates using mitochondrial loci (reviewed in Powell 1997), and there is also some general concordance with what little paleontological evidence is available for Drosophila (see [34]). We also used *D. pseudoobscura* and *D. persimilis* from the *D. obscura* group diverged for about 0.85 Mya [34]. These two species belong to the *Obscura* group that diverged 55 Mya from the melanogaster group [34], which brings about problems of saturation in *d*_*S*_ and gene expression differences [35]. Nevertheless, this species pair diverged for roughly the same amount of time as *D. simulans-D. sechellia*, a comparison of rates of protein evolution between the two species pairs will be useful to determine if acceleration in rates of evolution is commonly found among newly derived species.

### 2.3. Estimating Differences in Rates of *d*_*N*_ in relation to Speciation

**Figure 1:**
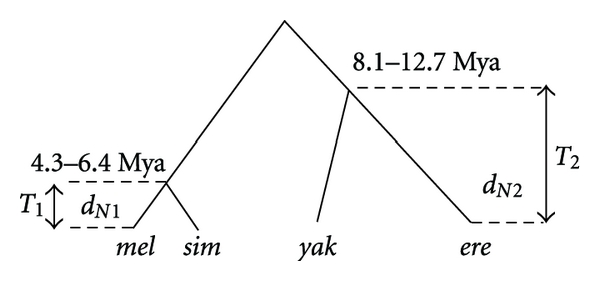
Schematic representation of rational implemented in our methodology. *d*_*N*1_: proportion of nonsynonymous substitutions in the more recently diverged species pair (diverged for time *T*_1_). *d*_*N*2_: proportion of nonsynonymous substitutions in species that have diverged for longer periods of time (*T*_2_).

According to the divergence times recently reported by Tamura et al. [34], *D. erecta* and *D. yakuba* have diverged for an estimated length of time that is ~2-3 times greater than the divergence time between *D. melanogaster* and *D. simulans*. Given the neutrally expected linear relationship between genetic divergence and time, we should therefore expect ~2-3 times greater divergence in genes between *D. yakuba* and *D. erecta* relative to *D. melanogaster* and *D. simulans*. We calculated the expected rate of molecular divergence (rate of nonsynonymous mutations per nonsynonymous sites *d*_*N*_ and rate of synonymous mutations per synonymous sites *d*_*S*_) using the relationship given below. Since our interest ultimately was to determine differences in rates of protein evolution between the different species pairs we focused more on *d*_*N*_. The ratio of nonsynonymous divergence (*d*_*N*_) between two-species pairs, a recently diverged species pair 1 and an older species pair 2, must be proportional to the ratio of their divergence times (*T*), such that:

(1) $$\log_{2}\;\left(\frac{T_{1}}{T_{2}}\right)=\log_{2}\;\left(\frac{d_{N1}}{d_{N2}}\right),$$

where, for a given species pair, *T*_1_ = divergence time of the more recently diverged pair and *T*_2_ = divergence time of the species with longer post-speciation divergence time. In our data, for example, *T*_1_ = 4.3–6.5 Mya (mel-sim) and *T*_2_ = 8.1–12.7 Mya (yak-ere), or, *T*_1_ = 0.4–0.6 Mya (sim-sec) and *T*_2_ = 8.1–12.7 Mya (yak-ere) (see Figure 1). We similarly calculated the ratio of synonymous divergence to species divergence time in all species pairs. Because of existing uncertainties in the divergence time estimates, particularly for the newer species [36, 37], we worked with the estimated *range of divergence times* for each of the species pairs by incorporating the published upper and lower limits of divergence time estimates from Tamura et al. [34]. For instance, in comparisons between *D. melanogaster*-*D. simulans* versus *D. yakuba*-*D. erecta* pairs, the upper divergence-time limit represents a scenario where *D. melanogaster* and *D. simulans* diverged 4.3 Mya and *D. yakuba* and *D. erecta* diverged 12.7 Mya. Similarly, the lower divergence time limit represents a scenario where *D. melanogaster* and *D. simulans* diverged 6.5 Mya, and *D. yakuba* and *D. erecta* diverged 8.1 Mya. When log_2_ (*d*_*N*1_/*d*_*N*2_) is plotted against log_2_ (*T*_1_/*T*_2_), genes evolving in a clock-like manner (i.e., *d*_*N*1_ = [*d*_*N*2_∗(*T*_1_/*T*_2_)]) would fall within these divergence time-ranges. Genes that fall above the divergence time-ranges indicate accelerated evolution in the newer species and those falling below the lower limit are considered to evolve slowly in the newly formed species or to have much higher rates of evolution in the older species lineage.

**Figure 2:**
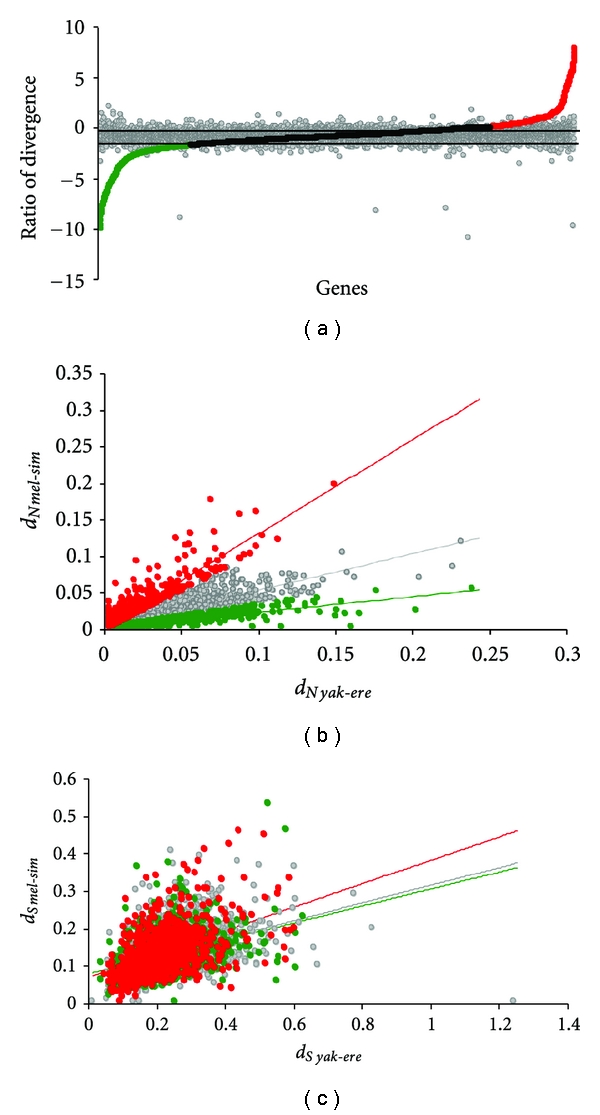
Distribution of ratio of divergence between *D. melanogaster-D. simulans* versus *D. yakuba-D. erecta*. (a) Distribution of molecular-divergence log_2_ (*d*_*N*1_/*d*_*N*2_) to divergence-time ratio log_2_ (*T*_1_/*T*_2_), between *D. melanogaster-D. simulans* and *D. yakuba-D. erecta*. Colored circles represent *d*_*N*_ ratios, and gray circles represent *d*_*S*_ ratios. The upper limit of the clockwise category is given by the log_2_ ratio of divergence times (6.5/8.1) and the lower limit by the log_2_ ratio 4.3/12.7. (b) Relationship of *d*_*N*_ between *D. melanogaster-D. simulans* and *D. yakuba-D. erecta*. The regression lines for each rate categories are also plotted. (c) Relationship of *d*_*S*_ between *D. melanogaster-D. simulans* versus *D. yakuba-D. erecta*; color codes: red: accelerated, black: clock-like, green: slow-rate categories, and grey: *d*_*S*_ ratios.

**Figure 3:**
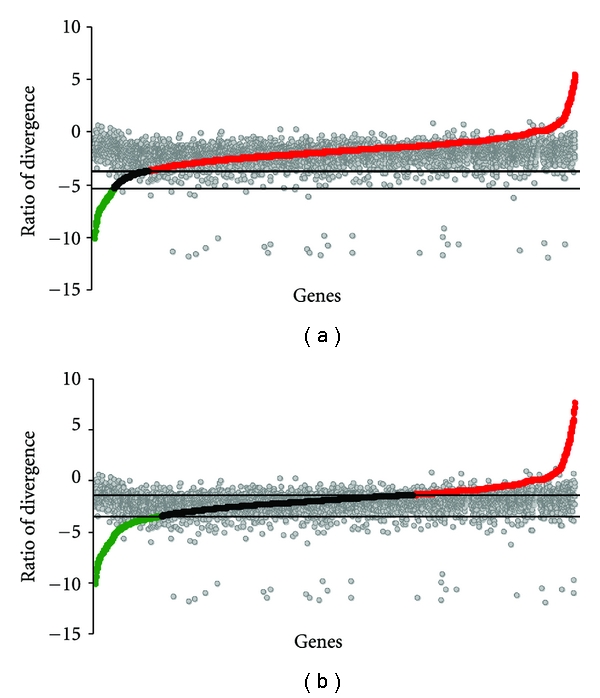
Distribution of ratio of divergence between *D. simulans-D. sechellia and D. yakuba-D. erecta*. (a) Distribution of ratio of amino acid replacement substitutions in separate clades log_2_ (*d*_*N*1_/*d*_*N*2_) to divergence time (according to Kliman et al. [33]) ratio log_2_ (*T*_1_/*T*_2_), *D. simulans-D. sechellia* and *D. yakuba-D. erecta*. (b) Re-evaluation of divergence times between these species. *d*_*N*_ ratios are plotted in colors, and *d*_*S*_ ratios are plotted in grey. Color codes: red circles accelerated evolution, black: clock-like, green: slow evolution categories, grey: *d*_*S*_ ratios.

Comparisons using the *D. simulans-D. sechellia* species pair grossly overestimated the number of genes in the accelerated rate category (94.89% of the genes fall under the accelerated category Figure 3(a)). This is an unlikely scenario given that the range of *d*_*S*_ falls well above the divergence times plotted in this graph. We believe that this is most likely due to a gross underestimation of the divergence time between *D. simulans* and *D. sechellia*. The phylogenetic relationships in the *simulans* triad (*D. simulans, D. mauritiana,* and *D. sechellia*) have been contentious and unresolved [35] and the sole source of recent divergence times estimates using molecular data comes from Kliman et al.'s study [33], which used a small number of genes. We took a very generalized approach to reevaluate the divergence time for the *D. simulans-D. sechellia* split using *d*_*S*_ estimated from our dataset (Figure 2) in order to replot Figure 3(a). Relative to nonsynonymous divergence, we expect the ratio of synonymous divergence-to-species divergence times for most loci to fall within the clock-like category for most species comparisons (as it did in the previous comparison). We therefore applied the *d*_*S*_ boundaries conforming to the clock-like category from *D. melanogaster-D. simulans* versus *D. yakuba-D. erecta* comparisons (Figure 2) to the *D. simulans-D. sechellia* species pair. We were able to arrive at a generalized estimate of 1.16–3.05 Mya for the *D. simulans-D. sechellia* split which appears more likely (Kumar 2007, pers comm.). We therefore employed a slightly more stringent approach by applying 95% confidence intervals to the existing divergence boundaries (using *d*_*S*_ ratios), which consequently incorporated previous outliers (Table 1).

### 2.4. Sequences and Rate Analyses

All sequences (*D. melanogaster, D. simulans, D. sechellia, D. yakuba, D. erecta, D. pseudoobscura*, and *D. persimilis*) were obtained from the recently sequenced genomes available on FlyBase (*Drosophila* 12 Genomes consortium 2007, http://www.flybase.org/). Sequences were aligned according to the corresponding protein alignment using CLUSTALW ver. 1.8 [38]. In order to remove any potential bias due to incomplete lineage sorting effects [39, 40], for each gene we compared the likelihood of trees differing in the placement of *D. yakuba* and *D. erecta* using PAML and restricted our analysis to genes for which the best tree involved *D. yakuba* and *D. erecta* as sister species. Pairwise estimates of *d*_*N*_ and *d*_*S*_ were determined using the program codeml in PAML [41]. Estimates of *d*_*N*_, *d*_*S*_, and *ω* along each lineage using branch site models and outputs of models M7 (neutral) versus M8 (positive selection) were also determined using PAML [42] and retrieved from a recent genome-wide analysis [43]. We were able to compute estimates of divergence to time ratio (see above) for 4843 orthologs between *D. melanogaster-D. simulans* versus and *D. yakuba-D. erecta*, and 4327 orthologs between *D. simulans-D. sechellia* versus *D. yakuba - D. erecta* comparisons as well as for 3988 genes from *D. pseudoobscura-D. persimilis* versus all melanogaster species pairs.

### 2.5. Classification of Genes according to Site of Expression

We classified genes according to their tissues of expression (testis, ovary, and head) by using the NCBI EST database (NCBI, http://www.ncbi.nlm.nih.gov/UniGene/). Genes that could not be classified into any tissue category were referred as unspecified. Based on EST data, we were able to classify tissue of expression for 3040 out of 4843 genes for comparisons involving *D. melanogaster-D. simulans* versus *D. yakuba-D. erecta*, 2686 genes for comparisons involving *D. simulans-D. sechellia* versus *D. yakuba-D. erecta* (Table S1).

## 3. Results

### 3.1. Accelerated Rates of Molecular Evolution in Newly Formed Species

Molecular divergence estimates, with respect to species divergence times, showed that protein coding genes fell into three distinct rate categories: (1) *accelerated evolution in younger species:* genes showing higher rates of molecular divergence in newly formed species relative to species diverged for longer lengths of time. (2) *clock-like evolution*: genes showing molecular divergence that corresponds to species divergence times, and (3) *slow evolution in younger species*: genes showing lower rates of molecular divergence in newly formed species in comparison to species with longer divergence times (Figure 2). Table 1 summarizes the fraction of genes that fall under each rate category for every species pair compared.

**Figure 4:**
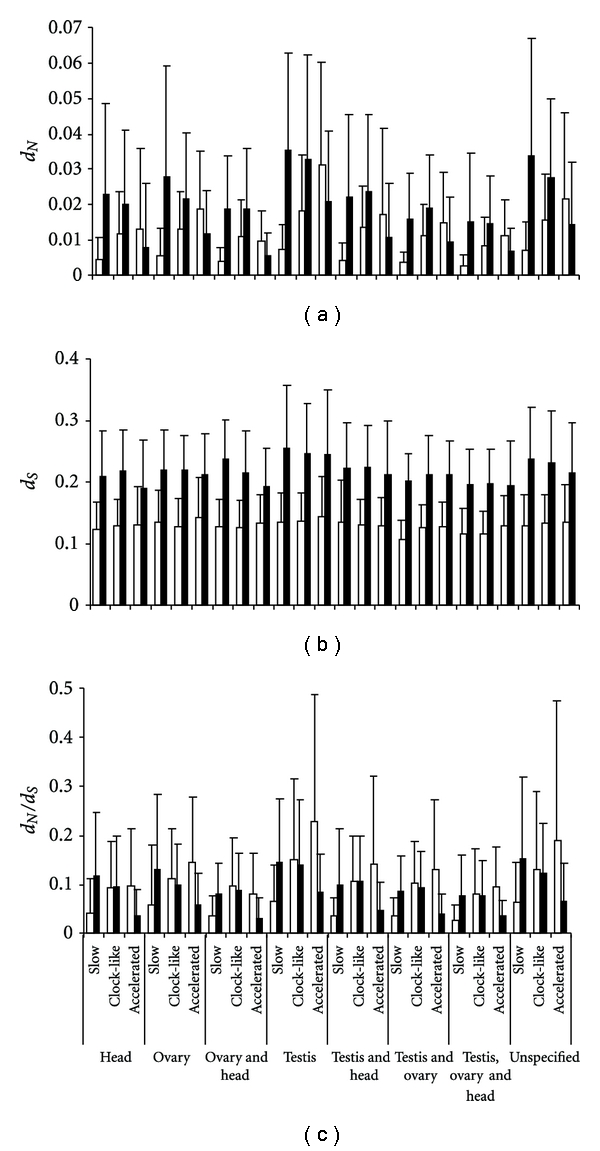
Relationship between rate category and tissue of expression. Distribution of (a) *d*_*N*_, (b) *d*_*S*_, and (c) *d*_*N*_/*d*_*S*_ between rate categories (accelerated, neutral, and slow) and between tissue classifications. White bars: *D. melanogaster-D. simulans*, and black bars: *D. yakuba-D. erecta*. The error bars represent the standard deviation.

**Figure 5:**
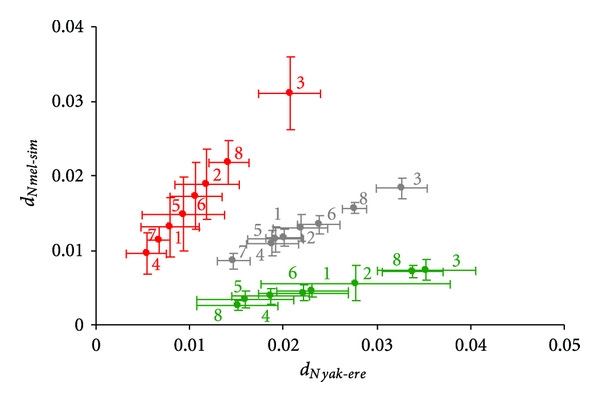
Relationship between evolutionary rates and tissue of expression. Relationship between *d*_*N* *D*.*melanogaster*-*D*.  *simulans*_ and *d*_*N* *D*.  *yakuba*-*D*.  *erecta*_ for gene classifications in each rate category: red: accelerated, grey: clock-like, green: slow. Tissue classifications: (1) head-specific; (2) ovary-specific; (3) testis-specific; (4) ovary and head; (5) ovary and testis; (6) testis and head; (7) Testis; ovary and head; (8) unknown. Error bars represent 95% confidence intervals.

Most genes in our dataset (61–74%) fell into the clock-like rate category in all comparisons, indicating that evolutionary trajectories of most genes are unaffected by speciation events and their rates remained constant (Table 1). A small but discernable fraction of genes (17–19%) showed signatures of speciation-related accelerated evolution. Nonsynonymous divergences in these genes were 2-3-fold higher in newly formed species compared to older species (Table 1, Figures 2 and 3). A plot of *d*_*N*_ and *d*_*S*_ estimates between *D. melanogaster*-*D. simulans* versus *D. yakuba*-*D. erecta* shows that the distribution of *d*_*N*_ estimates is quite distinct between the accelerated, clock-like, and slow-rate categories while the distribution of *d*_*S*_ estimates is not (Figures 2(b) and 2(c)). This quite clearly indicates that elevated proportions of *d*_*N*_ is not always accompanied by correspondingly elevated proportions of *d*_*S*_ in genes with evidence of accelerated evolution, which is generally a sign of selection driven changes. We also obtained similar results using protein divergence estimates instead of the ratios of nucleotide divergence data See files in Supplementery Material available online at doi: 10.4061/2011/595121. About 8–19% of protein coding genes that fell into the slow rate category showed extremely low levels of nonsynonymous divergence in newly formed species compared to older species (e.g., Avg *d*_*Nmel*-*sim*_ = 0.0057 ± 0.007, Avg *d*_*Nyak*-*ere*_ = 0.2276 ± 0.081, Figures 2, 4, and 5). This may be indicative of genes that have remained conserved in the evolution of the recently diverged species but that have diverged substantially with time in the older lineages. 

These results quite unambiguously indicate two important trends: firstly, despite variances associated with divergence estimates, the 2-3-fold higher nonsynonymous divergence in newly formed species compared to species with much longer divergence times clearly represents increases in rates of protein evolution either during or immediately after speciation. Secondly, accelerated rates of molecular evolution are most apparent in nonsynonymous divergence and not in synonymous divergence (see Table 1, Figures 2 and 4), a broadly accepted sign of selection driven changes.

### 3.2. Higher Representation of Sex-Related Genes in the Accelerated and Clock-Like Rate Categories

Identifying the range and, particularly, the kinds of loci in each rate category (accelerated, clock-like and slow) provides a starting point to broadly address the effects of demographic factors and selection during speciation. Demographic factors (drift, bottlenecks etc.) would affect a wide variety of loci whereas selection driven divergence would only affect specific functional classes of genes, such as sex-related genes which are expected to drive reproductive isolation in diverging populations [45, 46]. Due to lack of functional information for a large number of *Drosophila* genes, we used tissue of expression as a general and presumable indication of function (testis and ovary as reproductive tissues versus head, a presumably nonreproductive tissue). We tested the null hypothesis that genes expressed in each tissue-type are equally distributed within the accelerated, clock-like and slow rate categories. Because the expression data was determined using *D. melanogaster* data, only species from the *D. melanogaster* subgroup were analyzed. Given the long divergence time between the *Obscura* group and the melanogaster group (55 Mya), it is likely that patterns of gene expression may be radically different in *D. pseudoobscura* and *D. persimilis*. 

In the *D. melanogaster*-*D. simulans* versus *D. yakuba*-*D. erecta* comparison, a significantly higher proportion of testis-specific genes occupy the accelerated and clock-like rate categories compared to the slow-rate category (*χ*²  testis specific = 8.08 and 6.78, df = 1, and *P* = 0.0134 and 0.0277, respectively, and a Bonferroni correction was applied, Table 2, [44]). Genes expressed in all three tissues—testis, ovary and head mostly fall in the accelerated category compared to both slow and clock-like rate categories (*χ*²  testis, ovary-and-head, = 5.96 df = 1, *P* = 0.0439, *χ*² = 19.99, df = 1, *P* = 2.34 × 10^−5^, Bonferroni corrections were applied [44], Table 2). No significant differences were observed in the proportions of all other genes classes between rate categories. 

In the *D. simulans-D. sechellia/D. yakuba-D*. erecta comparison, a significantly small proportion of testis-specific genes fall into the accelerated category compared to clock-like and slow rate categories (**χ**² = 10.04 and 13.91, df = 1, *P* = 4.59 × 10^−3^ and 5.75 × 10^−4^, a Bonferroni correction was applied, Table 2).

### 3.3. “Persistence” of Rapid Evolution in Testis-Specific Genes over Time

That most testis-specific genes fall under the clock like rate category and only a small proportion fall in the accelerated rate category would appear to contradict earlier evidence that testis specific genes are in general evolving rapidly [20, 47, 48]. We hypothesized that a “persistence” of rapid rates of evolution in testis specific genes (rapid rates of evolution over time) will explain why testis-specific genes largely occupy the clock-like rate category. Pagel et al. [13] also found such persisting elevated rates of evolution in many lineages. Persistence of rapid rates of evolution can be verified if testis specific genes in both younger and older lineages have high substitution rates in both the accelerated and clock-like rate categories compared to other genes. To test this, we first looked for a global difference in *d*_*N*_ and *d*_*S*_ between tissue categories as well as between rate categories (Figure 5). We also performed more detailed analyses of differences in *d*_*N*_ and *d*_*S*_ between each tissue category using a Tuckey HSD test. Invariably, testis-specific genes show significantly higher *d*_*N*_ and *d*_*N*_/*d*_*S*_ compared to genes in all other tissues in both younger and older species pairs (*P* < 0.05 for both, Figures 4(a), 4(b), and 4(c)) (additional file 3). This implies that testis genes in the clock-like category are evolving faster than other genes but at a constant rate, whereas those in the accelerated rate category have experienced elevated rates of evolution in response to stronger selection during or after speciation. This is an important trend as it reveals a tempo of molecular evolution in the different classes of genes.

### 3.4. Genes Evolving under Positive Selection in the Accelerated, Clock-Like, and Slow Rate Categories

We compared the proportion of genes showing evidence of positive selection (*ω* > 1.0) among the different rate categories by implementing site models, branch-site and branch models in PAML [42]. Applying models M7 versus M8, none of the rate categories showed overrepresentation of genes evolving positively (slow versus accelerated, **χ**² = 0.88, df = 1, *P* > 0.05, accelerated, slow versus clock-like **χ**² = 0.93 and 2.46, df = 1, *P* > 0.05 and 0.35 after Bonferroni corrections, Table 3). Using the branch-site model however, we observed a significant over-representation of genes showing positive selection in *D. simulans* in the accelerated rate category compared to the clock-like and slow rate categories (**χ**² = 20.82 and 28.6, df = 1, *P* = 1.51 × 10^−5^ and 2.67 × 10^−7^, respectively after Bonferroni correction). Branch model tests also show a greater proportion of genes with foreground *ω* > background *ω* in the accelerated rate category in the *D. simulans* branch and the branch leading to the *D. melanogaster* clade (Table 3). A list of genes detected to be evolving under positive selection in each lineage can be found in additional file 4. Several genes involved in sensory stimuli, immune response, gametogenesis (spermatogenesis), transcription regulation, and hybrid incompatibilities (including *Hmr*, [21] are amongst the genes that show relatively large *ω* estimates. Population genomic study of six *D. simulans* strains compared to *D. melanogaster* [49] found significant evidence of directional selection in genes affecting reproduction or spermatogenesis. Among the 1270 genes that show evidence of adaptive evolution from Singh [46], 505 are found in our comparison of *D. melanogaster-D. simulans/D. yakuba-D. erecta*. Among the 505 genes, 360 fall in the clock-like category, 108 in the accelerated category and 37 fall in the slow rate category. We detect a significant enrichment of genes under positive selection among the clock-like category **χ**² = 7.25, df = 1, *P* = 7.09 × 10^−3^) while we found a significant paucity among the slow rate category (**χ**² = 40.05, df = 1, *P* = 2.48 × 10^−10^). No significant effect is observed for the genes classified as rapidly evolving in the younger species (**χ**² = 2.72, df = 1, *P* = 0.099).

### 3.5. Effect of Local Recombination Rates

Gene evolution may be influenced by their chromosomal location [50, 51] as well as by local recombination rates [43]. Using recombination rates in *D. melanogaster* computed by Hey and Kliman [52], we found significantly higher average recombination rates for genes in the accelerated rate category compared to the clock-like and slow categories in the *D. melanogaster-D. simulans/D. yakuba-D. erecta* comparison (Kruskal Wallis rank sum test, *P* < 2.2 × 10^−16^ and *P* = 0.0234, respectively, a Bonferroni correction was applied). There was no significant difference between the clock-like and slow rate categories (Kruskal Wallis rank sum test, *P* = 0.323, a Bonferroni correction was applied). The *D. simulans-D. sechellia/D. yakuba-D. erecta* comparison showed no such effect of recombination in all rate categories (Kruskal Wallis rank sum test, *P* = 0.67, 0.30 and for clock-like versus accelerated, accelerated versus slow, clock-like versus slow, respectively after Bonferroni correction).

## 4. Discussion

### 4.1. What Kinds of Genes Change during Speciation?

Despite recent evidence linking accelerated rates of molecular evolution to speciation events [12, 13], the kinds of protein coding genes that might experience accelerated rates of evolution during or immediately after speciation require investigation [15]. Are rates of evolution in all genes likely to be altered during or even after speciation? If not, what genes do show speciation associated changes? Answers to these questions will be crucial to understanding the mechanism(s) driving speciation and the molecular evolutionary consequences of speciation. 

This study analyzes the ratio of molecular evolutionary rates to the ratio of species divergence times, therefore clock-like evolution should not be taken as a sign of gradual evolution; it only implies a constant rate of evolution. This is exemplified by the large representation of testis-specific genes in the clock-like rate category, which are in fact evolving much faster than other genes in the same category. Furthermore, *Drosophila* is one of those groups that lack detailed fossil records and incomplete taxon sampling is an obvious but unavoidable problem in this, or any study of this nature. Apart from extinct taxa, undiscovered and undescribed taxa are an additional problem. We are also lacking the genome sequence of *D. mauritiana*, a member of the *D. simulans-D. sechellia-D. mauritiana* triad as well as the genome of *D. santomea* which recently split from *D. yakuba* [53]. These factors are bound to affect true assessments of speciation-related changes in rates of molecular evolution. However, within the currently accepted phylogenetic network of the melanogaster subgroup, our study provides an approach to identify genes that change during speciation and out results report the fraction and identity of genes in the *Drosophila* transcriptome that show signatures of accelerated speciation-related changes that can be further investigated.

### 4.2. Evidence for Accelerated Evolution

In our study, evidence of speciation-related accelerated evolution is detected in a small but discernable fraction of protein coding genes. This corresponds to what Pagel et al. [13] found across taxa. But what is striking is that the acceleration is most apparent in nonsynonymous divergence. That d_S_ in most genes fall under the clock-like category as opposed to d_N_ is not entirely surprising, as we expect *d*_*N*_ changes to be more sensitive to selection and may therefore show higher variance. Nevertheless, the 2-3 fold higher estimates of d_N_ in the recently diverged species compared to species with much longer divergence times is a strict deviation from clock-like evolution despite the variance that may be involved. More importantly, our survey reveals that the more recently diverged species (*D. simulans*-*D. sechellia*) have a slightly greater proportion of genes that show accelerated rates of evolution (Table 1), further strengthening the case for a causal link between acceleration in rates of molecular evolution and speciation.

### 4.3. Evidence for Persistent Rapid Evolution in Sex Genes

Sexually reproducing organisms are influenced both by natural and sexual selection. Widespread rapid evolution of sex-related genes in *Drosophila* genomes [18] imply that regardless of how speciation occurred, sex and reproduction related genes that are causally involved in establishing reproductive isolation would be constantly under strong selection [46]. Therefore we would not expect to find an over-representation of sex-related genes in the accelerated rate category alone. This is specifically illustrated by the persistence of rapid rates of evolution in testis specific genes in our study. In addition, testis-specific genes and genes expressed in all three tissues are over-represented in the accelerated and clock-like rate category in the *D. melanogaster*-*D. simulans* pair (Table 2). But in the more recently diverged *D. simulans*-*D. sechellia* pair, only testis-specific genes are overrepresented in the clock-like and slow rate categories (Table 2). These results support a scenario where sex-related genes are under constant but higher selective pressure. 

### 4.4. Factors Driving Accelerated Evolution during Speciation

We find no evidence of widespread positive selection in the accelerated rate category. The higher proportion of nonsynonymous divergence we observe in the accelerated rate category cannot be ruled out due to relaxed constraint or accumulation of slightly deleterious mutations [16, 54, 55]. The only tenable links to acceleration that we find appear to be influenced by local recombination rates (higher in the *D. melanogaster*-*D. simulans* comparison but lower in the *D. simulans*-*D. sechellia* comparison) and a relatively small proportion of genes with lineage specific positive selection (Table 3). Variation in recombination rates between species can be an important force driving divergence, as new allele combinations can be produced at different rates within species [56]. Rate of recombination also appears to be positively correlated with levels of polymorphism and high polymorphism would be expected to correlate with levels of divergence [57]. Nonetheless, sex-related genes involved in gametogenesis, hybrid incompatibilities as well as genes involved in metabolism and sensory functions do show evidence of positive selection. This implies a loci-specific role of sex genes and sexual selection in speciation along with some ecological specialization. Our data therefore indicates that acceleration in rates of evolution is not purely a consequence of strong selection but most likely a combination of factors, such as demographic processes as well as some form of selection (a few genes in the accelerated category are evolving positively, Table 3). These data are interesting particularly in *D. sechellia*, which is a relatively young species and one that is likely to have undergone founder speciation (see [33]).

According to the founder effect model of speciation [3], speciation occurs as a consequence of major demographic processes in which bottlenecks play an important role. In such a case, a large number of loci involved in a wide range of functions would be affected (but see [58] for a more recent critical analysis of bottlenecks and speciation). Our results as well as those of Pagel et al. [13] do not support the notion of a genetic revolution as a consequence of speciation; the range of loci affected during speciation is rather limited. However, loci affecting a wide range of functions show accelerated rates of evolution (Figures 1, 2, and 3, Tables 1 and 2). This supports a scenario where, as founding populations adapt to new ecological niches the evolution of modified or new behaviors affecting sex and reproduction as well as other ecological adaptations can occur rapidly [59–62]. *D. sechellia* has evolved an intricate ecological relationship with its plant host *Morinda citrifolia*, on which *D. sechellia* females oviposit their eggs [63]. Compounds in the pulp of the immature *M. citrifolia* fruit are toxic to other species of the melanogaster subgroup but not to *D. sechellia* larve that feed and grow on it [63]. A large proportion of head-expressed genes show accelerated evolution in the *D. simulans*-*D. sechella* pair, which might reflect rapid behavioral and sensory modifications that occurred during the host-plant specialization between *D. sechellia* and *M. citrifolia*. Genes involved in sensory perception (*Gr2a, Gr21a, Gr43a, Obp50a, Or67d, king-tubby, CG32683*), sensory organ development (*amos, Brd, mib1, Oseg1, Poxn*), detoxification (*kraken*), metabolism, and oogenesis (*cup, retn, kel, spir, Tm1*) found to be involved in host specialization by Dworkin and Jones [64] are among those that show evidence of accelerated speciation-related rates of evolution in our study. 

The *D. sechellia* data demonstrates the importance of founder-effect and subsequent ecological divergence in driving speciation and as a consequence, causing elevated rates of evolution of relevant protein coding genes associated with the speciation event. Such cases of speciation driven by founder-effect and subsequent resource specialization are not uncommon in Drosophila [45, 59, 65]. *D. sechellia, D. mauritiana* and *D. santomea* (a recently described sibling species to *D. yakuba*) are all insular species within the melanogaster subgroup. Among these, *D. sechellia and D. santomea* show resource specialization having evolved special ecological relationships of utilizing specific host plants either for food or oviposition [53, 63–65], while their parent lineages do not. Such species-specific ecological adaptations are likely to influence accelerated evolution in the newly formed species but not necessarily in the parent lineages. Our study support this reasoning; we find ~400 genes in the accelerated rate category in the *D. simulans* versus *D. sechellia* comparison; all of which fall in the clock-like rate category in the *D. melanogaster* versus *D. simulans* comparison (see additional file 5). Many of these genes, as mentioned above, have been found to be involved in host-plant specialization [64]. Further detailed study of such genes between *D. sechellia* and *D. simulans*, as well as the inclusion of *D. santomea* genome (when completed) in a similar study should shed some light on our claim. However, it should be noted that while *D. sechellia* is isolated from *D. simulans, D. santomea* and its sibling species *D. yakuba* are sympatric on the Island of San Tome and there is substantial divergence of sexual traits between the two species [53, 66–69]. We might therefore expect to see higher proportions of sex-related genes as well as genes involved in ecological specializations to show signatures of accelerated evolution between *D. santomea* and *D. yakuba*, much like what we observed between the sympatric *D. melanogaster* and *D. simulans* in our study.

## 5. Conclusion

Signatures of speciation-related accelerated rates of evolution are detected in all newly evolved species analyzed in this study. This is not observed as a widespread genomic signature but is restricted to a fraction of the genome that affects widely different functions. The range and kinds of loci in this rapidly evolving fraction identified in this study complements the recently reported punctuational effect observed across a variety of taxa [13, 15] and improves our focus of studying the genetics of speciation. Population genetic studies of these ‘candidate' genes can establish the nature of selective forces driving their elevated rates of evolution. In our case, we find a demography-selection driven effect. Testis specific genes and sex-related genes show persistently high rates of evolution, indicating that sexual selection is a constant pressure in diverging and established populations. In the *D. simulans-D. sechellia* pair, our data reinforce previous evidence that founder effect and ecological specialization played important roles in their speciation process. Our results support the growing appreciation that speciation is often driven by a combination of demographic fluctuations, ecological adaptation, and sexual selection and can seldom be attributed to one single important factor [45, 70].

## Supplementary Material

Additional file 1: Comparison between D. melanogaster – D. simulans / D. yakuba – D. erecta.
Additional file 2: Distribution of protiendivergence log2 (P1/P2) to divergencetime ratio log2 (T1/T2), between D. melanogaster D. simulans vs. D. yakuba D. erecta (Supplementary Table 1). The upper limit of the clockwise category is given by the log2 ratio of of divergence times (6.5/8.1) and the lower limit by the log2 ratio 4.3/12.7. Color codes: Red circlesaccelerated evolution, Grey – clocklike, green slow evolution categories.
Additional file 3: Dendrogram of average *ω* estimates of genes falling under slow, clock-like and accelerated rate categories. Branch lengths represent the proportion of genes with *ω* values greater than background *ω* values. P-values for the different tests were corrected using a Bonferroni correction prior to the comparisons. mel: D. melanogaster, sim: D. simulans, sec: D. sechellia, simsecmel: branch leading to the D. melanogaster clade, yak: D. yakuba, ere: D. erecta, yakere, branch leading to the D. yakuba clade, ana: D. ananassae.
Additional file 4: Genes with evidence of positive selection using site models (M8 vs M7) from PAML.
Supplementary data 5: Comparison of gene classification in between the D. simulans - D. Sechellia / D. yakuba – D. erecta and D. simulans – D. melanogaster / D. yakuba – D. erecta analyses.

## Figures and Tables

**Table 1:** Number and (percent) of genes falling under accelerated, clock-like, and slow-rate categories in recently diverged species when compared to older species pairs. (a) Analysis using the published divergence boundaries according to Kliman et al. [33] for *D. simulans* and *D. sechellia*, and Tamura et al. [34] for *D. melanogaster-D. simulans* and *D. yakuba-D. erecta*. (b) Analysis using the revised divergence time estimates for *D. simulans* and *D. sechellia*. (c) Analysis applying the 95% confidence interval boundaries on revised divergence estimates.

	Rate Categories		
	Slow	Clock-like	Accelerated
(a)			
*mel-sim *versus* yak-ere *			
*d*_*N*_	1096 (22.63)	2251 (46.48)	1496 (30.89)
*d*_*S*_	348 (7.19)	3628 (74.91)	867 (17.90)
*sim-sec *versus* yak-ere *			
*d*_*N*_	173 (3.99)	314 (7.26)	3840 (88.75)
*d*_*S*_	37 (0.86)	184 (4.25)	4106 (94.89)

(b)			
*sim-sec *versus* yak-ere *			
*d*_*N*_	594 (13.73)	2275 (52.58)	1458 (33.69)
*d*_*S*_	311 (7.19)	3241 (74.90)	775 (17.91)

(c)			
*mel-sim *versus* yak-ere *			
*d*_*N*_	963 (19.89)	2996 (61.86)	884 (18.25)
*d*_*S*_	242 (5.00)	4359 (90.00)	242 (5.00)
*sim-sec *versus* yak-ere *			
*d*_*N*_	469 (10.84)	3007 (69.49)	851 (19.67)
*d*_*S*_	216 (5.00)	3895 (90.00)	216 (5.00)

*pse-per *versus* sim-sec *			
*d*_*N*_	327 (8.20)	2962 (74.27)	699 (17.53)
*d*_*S*_	199 (5)	3590 (90)	199 (5)

**Table 2:** Number of genes present in each rate category when comparing *D. melanogaster-D. simulans* to *D. yakuba-D. erecta* and *D. simulans-D. sechellia* and to *D. yakuba-D. erecta*. Under brackets are the proportion of genes within tissue and the proportion within the rate category. Enrichment within a category was tested using *χ* ^2^, test and the Bonferroni corrections were applied [44].

Tissue	Accelerated	Clock-like	Slow	Total
*D. melanogaster-D. simulans *versus* D. yakuba-D. erecta *				

H	129 (17.18); (14.59)	445 (60.59); (14.85)	177 (22.63); (18.38)	**751 (15.51)**
O	47 (20.26); (5.32)	147 (63.79); (4.91)	38 (15.95); (9.95)	**232 (4.79)**
OH	35 (15.70); (3.96)	138 (63.68); (4.61)	50 (20.62); (5.19)	**223 (4.6)**
T	142 (20.67); (16.06)	440 (65.50); (14.69)	105 (14.70); (10.90)	**687 (14.19)**
TH	114 (20.36); (12.90)	357 (64.46); (11.92)	89 (15.72); (9.24)	**560 (11.56)**
TO	32 (20.65); (3.62)	99 (64.52); (3.30);	24 (14.84); (2.49)	**155 (3.2)**
TOH	116 (21.17); (13.12)	231 (55.97); (7.71)	80 (17.57); (8.31)	**427 (8.82)**
un	269 (14.88); (30.43)	1139 (64.66); (38.02)	400 (20.79); (41.54)	**1808 (37.33) **

Total	**884 (18.25)**	**2996 (61.86)**	**963 (19.89)**	**4843**

* D. simulans-D. sechellia versus D. yakuba-D. erecta*				

H	149 (21.94); (17.51)	458 (67.45); (15.23)	72 (10.6); (15.35)	**679 (15.69)**
O	32 (16.24); (3.76)	146 (74.11); (4.86)	19 (9.64); (4.05)	**197 (4.55)**
OH	48 (32.65); (5.64)	130 (65.99); (4.32)	19 (9.64); (4.05)	**197 (4.55)**
T	101 (16.13); (11.87)	427 (68.21); (14.20)	98 (15.65); (20.90)	**626 (14.47)**
TH	101 (19.65); (11.87)	363 (70.62); (12.07)	50 (9.73); (10.67)	**514 (11.88)**
TO	26 (19.55); (3.06)	99 (74.44); (3.29)	8 (6.02); (1.71)	**133 (3.07)**
TOH	77 (22.65); (9.05)	217 (63.82); (7.22)	46 (13.53); (9.81)	**340 (7.86)**
un	317 (19.32); (37.25)	1167 (71.11); (38.81)	157 (9.77); (33.48)	**1641 (37.92)**

Total	**851 (19.67)**	**3007 (69.49)**	**469 (10.84)**	**4327**

**Table 3:** Comparison of the proportion of genes showing evidence of positive selection under the site model, branch-site model, and branch model between slow (*n* = 963), clock-like (*n* = 2996), and accelerated (*n* = 884) categories. *P* values for the different tests were corrected using a Bonferroni correction prior to the comparisons. *mel: D. melanogaster, sim: D. simulans, sec: D. sechellia, simsecmel*: branch leading to the *D. melanogaster clade, yak: D. yakuba, ere: D. erecta, and yakere*: branch leading to the *D. yakuba* clade.

Model	Slow	Clock-like	Accelerated
Site model			
*M7/M8 *	6	40	9

Branch-site model			
*mel *	0	8	0
*sim *	5	51	39
*sec *	10	29	9
*simsecmel *	3	6	3
*yakere *	3	4	0

Branch model			
*mel *	0	6	6
*sim *	0	3	24
*sec *	1	13	3
*simsecmel *	1	59	80
*yak *	20	20	0
*ere *	36	26	0
*yakere *	68	83	0

## References

[1] Eldredge N, Gould SJ (1972). Punctuated equilibria: an alternative to phyletic gradualism. *Models in Paleobiology*.

[2] Gould SJ, Eldredge N (1993). Punctuated equilibrium comes of age. *Nature*.

[3] Barraclough TG, Vogler AP, Harvey P (1998). Revealing the factors that promote speciation. *Philosophical Transactions of the Royal Society B*.

[4] Jackson JBC, Cheetham AH (1999). Tempo and mode of speciation in the sea. *Trends in Ecology and Evolution*.

[5] Barraclough TG, Savolainen V (2001). Evolutionary rates and species diversity in flowering plants. *Evolution*.

[6] Benton MJ, Pearson PN (2001). Speciation in the fossil record. *Trends in Ecology and Evolution*.

[7] Hunt G (2007). The relative importance of directional change, random walks, and stasis in the evolution of fossil lineages. *Proceedings of the National Academy of Sciences of the United States of America*.

[8] Avise JC, Ayala FJ (1975). Genetic change and rates of cladogenesis. *Genetics*.

[9] Avise JC, Ayala FJ (1976). Genetic differentiation in speciose versus depauperate phylads: evidence from the California minnows. *Evolution*.

[10] Avise JC (1977). Is evolution gradual or rectangular? Evidence from living fishes. *Proceedings of the National Academy of Sciences of the United States of America*.

[11] Mindell DP, Sites JS, Graur D (1989). Speciation evolution: a phylogenetic test with allozymes in Sceloporus (Reptilia). *Cladistics*.

[12] Webster AJ, Payne RJH, Pagel M (2003). Molecular phylogenies link rates of evolution and speciation. *Science*.

[13] Pagel M, Venditti C, Meade A (2006). Large punctuational contribution of speciation to evolutionary divergence at the molecular level. *Science*.

[14] Leitch IJ, Beaulieu JM, Cheung K, Hanson L, Lysak MA, Fay MF (2007). Punctuated genome size evolution in Liliaceae. *Journal of Evolutionary Biology*.

[15] Venditti C, Pagel M (2010). Speciation as an active force in promoting genetic evolution. *Trends in Ecology and Evolution*.

[16] Arbiza L, Dopazo J, Dopazo H (2006). Positive selection, relaxation, and acceleration in the evolution of the human and chimp genome. *PLoS Computational Biology*.

[17] Savard J, Tautz D, Lercher MJ (2006). Genome-wide acceleration of protein evolution in flies (Diptera). *BMC Evolutionary Biology*.

[18] Clark AG, Eisen MB, Smith DR (2007). Evolution of genes and genomes on the *Drosophila* phylogeny. *Nature*.

[19] Panhuis TM, Clark NL, Swanson WJ (2006). Rapid evolution of reproductive proteins in abalone and *Drosophila*. *Philosophical Transactions of the Royal Society B*.

[20] Haerty W, Jagadeeshan S, Kulathinal RJ (2007). Evolution in the fast lane: rapidly evolving sex-related genes in *Drosophila*. *Genetics*.

[21] Barbash DA, Awadalla P, Tarone AM (2004). Functional divergence caused by ancient positive selection of a *Drosophila* hybrid incompatibility locus. *PLoS Biology*.

[22] Brideau NJ, Flores HA, Wang J, Maheshwari S, Wang XU, Barbash DA (2006). Two Dobzhansky-Muller Genes interact to cause hybrid lethality in *Drosophila*. *Science*.

[23] Masly JP, Jones CD, Noor MAF, Locke J, Orr HA (2006). Gene transposition as a cause of hybrid sterility in *Drosophila*. *Science*.

[24] Presgraves DC, Balagopalan L, Abmayr SM, Orr HA (2003). Adaptive evolution drives divergence of a hybrid inviability gene between two species of *Drosophila*. *Nature*.

[25] Ting CT, Tsaur SC, Wu ML, Wu CI (1998). A rapidly evolving homeobox at the site of a hybrid sterility gene. *Science*.

[26] Wittbrodt J, Adam D, Malitschek B (1989). Novel putative receptor tyrosine kinase encoded by the melanoma-inducing Tu locus in *Xiphophorus*. *Nature*.

[27] Zigler KS, McCartney MA, Levitan DR, Lessios HA (2005). Sea urchin bindin divergence predicts gamete compatibility. *Evolution*.

[28] Orr HA (2005). The genetic basis of reproductive isolation: insights from *Drosophila*. *Proceedings of the National Academy of Sciences of the United States of America*.

[29] Swanson WJ, Vacquier VD (2002). The rapid evolution of reproductive proteins. *Nature Reviews Genetics*.

[30] Metz EC, Palumbi SR (1996). Positive selection and sequence rearrangements generate extensive polymorphism in the gamete recognition protein bindin. *Molecular Biology and Evolution*.

[31] Zigler KS, Lessios HA (2003). Evolution of bindin in the pantropical sea urchin Tripneustes: comparisons to bindin of other genera. *Molecular Biology and Evolution*.

[32] Artieri CG, Haerty W, Singh RS (2007). Association between levels of coding sequence divergence and gene misregulation in *Drosophila* male hybrids. *Journal of Molecular Evolution*.

[33] Kliman RM, Andolfatto P, Coyne JA (2000). The population genetics of the origin and divergence of the *Drosophila simulans* complex species. *Genetics*.

[34] Tamura K, Subramanian S, Kumar S (2004). Temporal patterns of fruit fly (*Drosophila*) evolution revealed by mutation clocks. *Molecular Biology and Evolution*.

[35] Zhang Y, Sturgill D, Parisi M, Kumar S, Oliver B (2007). Constraint and turnover in sex-biased gene expression in the genus *Drosophila*. *Nature*.

[36] Kumar S, Filipski A, Swarna V, Walker A, Hedges SB (2005). Placing confidence limits on the molecular age of the human-chimpanzee divergence. *Proceedings of the National Academy of Sciences of the United States of America*.

[37] Rodríguez-Trelles F, Tarrío R, Ayala FJ (2002). A methodological bias toward overestimation of molecular evolutionary time scales. *Proceedings of the National Academy of Sciences of the United States of America*.

[38] Thompson JD, Higgins DG, Gibson TJ (1994). CLUSTAL W: improving the sensitivity of progressive multiple sequence alignment through sequence weighting, position-specific gap penalties and weight matrix choice. *Nucleic Acids Research*.

[39] Pollard DA, Iyer VN, Moses AM, Eisen MB (2006). Widespread discordance of gene trees with species tree in *Drosophila*: evidence for incomplete lineage sorting. *PLoS Genetics*.

[40] Wong A, Jensen JD, Pool JE, Aquadro CF (2007). Phylogenetic incongruence in the *Drosophila melanogaster* species group. *Molecular Phylogenetics and Evolution*.

[41] Yang Z (1997). PAML: a program package for phylogenetic analysis by maximum likelihood. *Computer Applications in the Biosciences*.

[42] Yang Z, Nielsent R (2002). Codon-substitution models for detecting molecular adaptation at individual sites along specific lineages. *Molecular Biology and Evolution*.

[43] Larracuente AM, Sackton TB, Greenberg AJ (2008). Evolution of protein-coding genes in *Drosophila*. *Trends in Genetics*.

[44] Holm S (1969). A simple sequentially rejective multiple test procedure. *Scandinavian Journal of Statistics*.

[45] Coyne JA, Orr AH (2004). *Speciation*.

[46] Singh RS, Singh RS, Krimbas C (2000). Toward a unified theory of speciation. *Evolutionary Genetics from Molecules to Morphology*.

[47] Civetta A, Singh RS (1995). High divergence of reproductive tract proteins and their association with postzygotic reproductive isolation in *Drosophila melanogaster* and *Drosophila virilis* group species. *Journal of Molecular Evolution*.

[48] Jagadeeshan S, Singh RS (2005). Rapidly evolving genes of *Drosophila*: differing levels of selective pressure in testis, ovary, and head tissues between sibling species. *Molecular Biology and Evolution*.

[49] Begun DJ, Holloway AK, Stevens K (2007). Population genomics: whole-genome analysis of polymorphism and divergence in *Drosophila simulans*. *PLoS Biology*.

[50] Baines JF, Sawyer SA, Hartl DL, Parsch J (2008). Effects of X-linkage and sex-biased gene expression on the rate of adaptive protein evolution in *Drosophila*. *Molecular Biology and Evolution*.

[51] Singh ND, Larracuente AM, Clark AG (2008). Contrasting the efficacy of selection on the X and autosomes in drosophila. *Molecular Biology and Evolution*.

[52] Hey J, Kliman RM (2002). Interactions between natural selection, recombination and gene density in the genes of *Drosophila*. *Genetics*.

[53] Lachaise D, Harry M, Solignac M, Lemeunier F, Benassi V, Cariou ML (2000). Evolutionary novelties in islands: *Drosophila santomea*, a new melanogaster sister species from São Tomé. *Proceedings of the Royal Society B*.

[54] Charlesworth J, Eyre-Walker A (2007). The other side of the nearly neutral theory, evidence of slightly advantageous back-mutations. *Proceedings of the National Academy of Sciences of the United States of America*.

[55] Ho SYW, Phillips MJ, Cooper A, Drummond AJ (2005). Time dependency of molecular rate estimates and systematic overestimation of recent divergence times. *Molecular Biology and Evolution*.

[56] Butlin RK (2005). Recombination and speciation. *Molecular Ecology*.

[57] Kulathinal RJ, Bennett SM, Fitzpatrick CL, Noor MAF (2008). Fine-scale mapping of recombination rate in *Drosophila* refines its correlation to diversity and divergence. *Proceedings of the National Academy of Sciences of the United States of America*.

[58] Nei M (2005). Bottlenecks, genetic polymorphism and speciation. *Genetics*.

[59] Carson HL (1987). The genetic system, the deme, and the origin of species. *Annual Review of Genetics*.

[60] Losos JB, Warheit KI, Schoener TW (1997). Adaptive differentiation following experimental island colonization in Anolis lizards. *Nature*.

[61] Losos JB, Schoener TW, Langerhans RB, Spiller DA (2006). Rapid temporal reversal in predator-driven natural selection. *Science*.

[62] Thompson JN (1998). Rapid evolution as an ecological process. *Trends in Ecology and Evolution*.

[63] R’Kha S, Capy P, David JR (1991). Host-plant specialization in the *Drosophila melanogaster* species complex: a physiological, behavioral, and genetical analysis. *Proceedings of the National Academy of Sciences of the United States of America*.

[64] Dworkin I, Jones CD (2009). Genetic changes accompanying the evolution of host specialization in Drosophila sechellia. *Genetics*.

[65] Cariou ML, Silvain JF, Daubin V, Da Lage JL, Lachaise D (2001). Divergence between *Drosophila santomea* and allopatric or sympatric populations of *D. yakuba* using paralogous amylase genes and migration scenarios along the Cameroon volcanic line. *Molecular Ecology*.

[66] Moehring AJ, Llopart A, Elwyn S, Coyne JA, Mackay TFC (2006). The genetic basis of prezygotic reproductive isolation between *Drosophila santomea* and *D. yakuba* due to mating preference. *Genetics*.

[67] Moehring AJ, Llopart A, Elwyn S, Coyne JA, Mackay TFC (2006). The genetic basis of postzygotic reproductive isolation between *Drosophila santomea* and *D. yakuba* due to hybrid male sterility. *Genetics*.

[68] Coyne JA, Kim SY, Chang AS, Lachaise D, Elwyn S (2002). Sexual isolation between two sibling species with overlapping ranges: *Drosophila santomea* and *Drosophila yakuba*. *Evolution*.

[69] Blyth JE, Lachaise D, Ritchie MG (2008). Divergence in multiple courtship song traits between *Drosophila santomea* and *D. yakuba*. *Ethology*.

[70] Ritchie MG (2007). Evolution: feathers, females, and fathers. *Science*.

