# Supplementary material for: Is Speciation Accompanied by Rapid Evolution? Insights from Comparing Reproductive and Nonreproductive Transcriptomes in Drosophila
Source: Int J Evol Biol. 2011 Aug 22;2011:595121. doi: 10.4061/2011/595121 (PMC3159995; doi:10.4061/2011/595121)

Additional file 2. Distribution of protien-divergence log2 (P1/P2) to divergence-time ratio log2 (T1/T2), between *D. melanogaster-D. simulans* vs. *D. yakuba -D. erecta* (Supplementary Table 1). The upper limit of the clock-wise category is given by the log2 ratio of of divergence times (6.5/8.1) and the lower limit by the log2 ratio 4.3/12.7. Color codes: Red circles- accelerated evolution, Grey – clock-like, green - slow evolution categories.

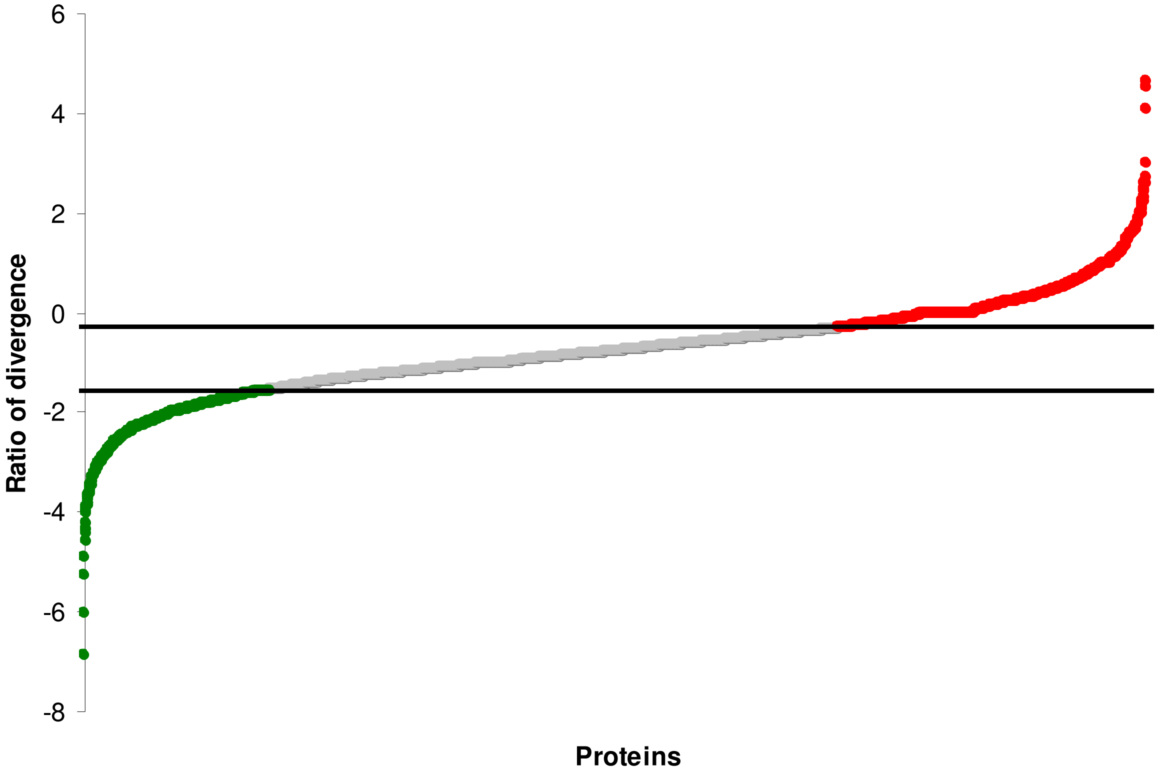

Supplement: Supplementary file 2 [file 595121.f2.pdf]
