# Supplementary material for: Is Speciation Accompanied by Rapid Evolution? Insights from Comparing Reproductive and Nonreproductive Transcriptomes in Drosophila
Source: Int J Evol Biol. 2011 Aug 22;2011:595121. doi: 10.4061/2011/595121 (PMC3159995; doi:10.4061/2011/595121)

Additional file 3. Dendrogram of average ω estimates of genes falling under slow, clock-like and accelerated rate categories. Branch lengths represent the proportion of genes with ω values greater than background ω values. P-values for the different tests were corrected using a Bonferroni correction prior to the comparisons. mel: *D. melanogaster*, sim: *D. simulans*, sec: *D. sechellia*, simsecmel: branch leading to the *D. melanogaster* clade, yak: *D. yakuba*, ere: *D. erecta*, yakere, branch leading to the *D. yakuba* clade, ana: *D. ananassae*.

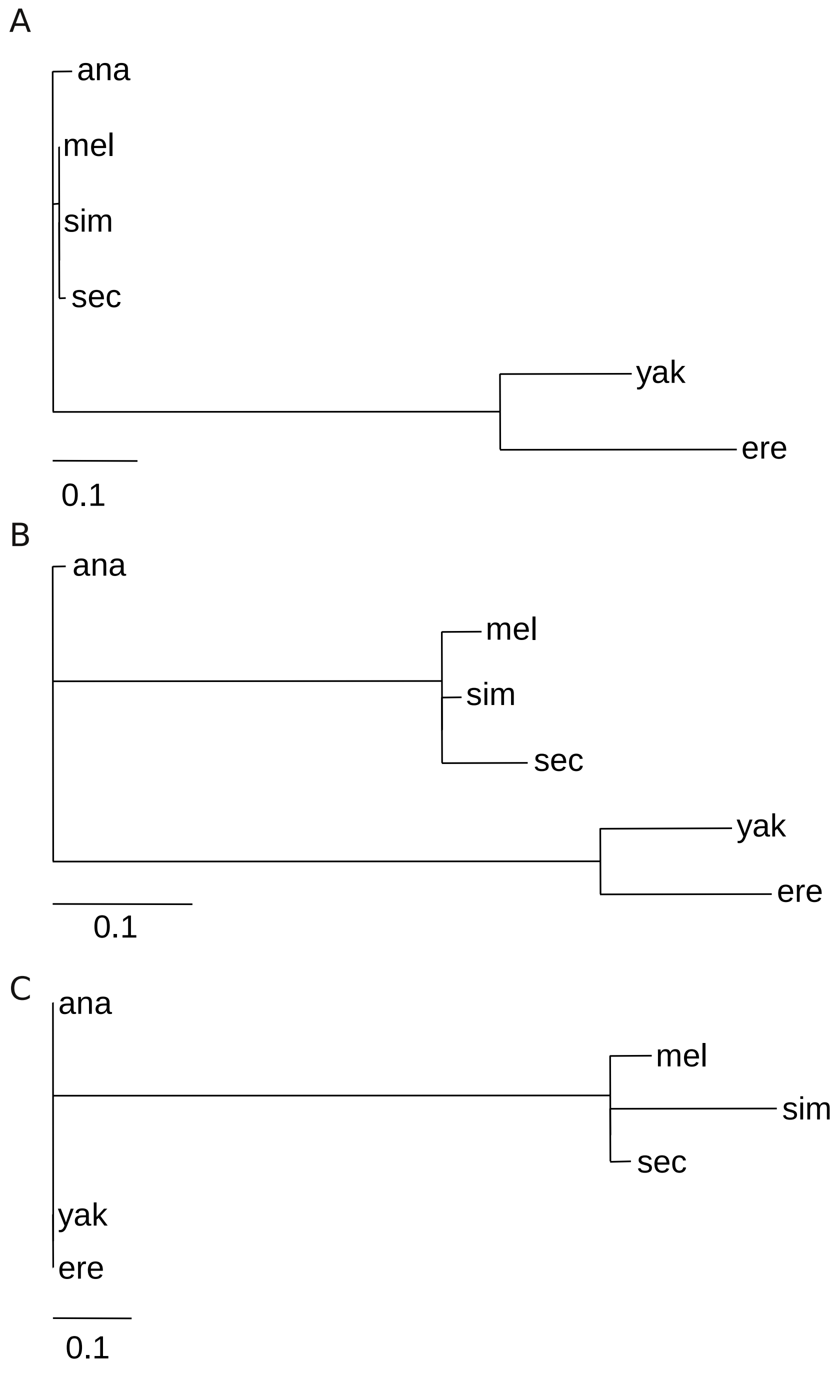

Supplement: Supplementary file 3 [file 595121.f3.pdf]
